# Antimicrobials in polymethylmethacrylate: from prevention to prosthetic joint infection treatment: basic principles and risk of resistance

**DOI:** 10.1186/s42836-023-00166-7

**Published:** 2023-03-02

**Authors:** Marta Sabater-Martos, Miguel A. Verdejo, Laura Morata, Ernesto Muñoz-Mahamud, Ernesto Guerra-Farfan, Juan C. Martinez-Pastor, Alex Soriano

**Affiliations:** 1 Department of Orthopedics and Traumatology, Hospital Clínic of Barcelona, Carrer Villarroel 170, 08036 Barcelona, Spain; 2 Department of Infectious Diseases, Hospital Clínic of Barcelona, Carrer Villarroel 170, 08036 Barcelona, Spain; 3 Department of Orthopedics and Traumatology, Hospital Vall d’Hebron of Barcelona, Passeig de la Vall d’Hebron 119, 08035 Barcelona, Spain; 4 University of Barcelona, CIBERINF, Carrer Casanova 143, 08036 Barcelona, Spain

**Keywords:** Antibiotic-loaded bone cement, Pharmacokinetics, Antibiotic prophylaxis, Spacers, Antibiotic resistance

## Abstract

**Background:**

Excellent revisions about antibiotic-loaded bone cement (ALBC) have been recently published. In the present article, we review the principles and limitations of local antibiotic delivery in the context of recent advances in the pathogenesis of prosthetic joint infections (PJI), with particular attention paid to the potential association between ALBC and antimicrobial resistance.

**Main body:**

Recalcitrance of PJI is related to the ability of pathogens to adapt to particular environments present in bone tissue and protect themselves from host immunity in different ways. Accordingly, delivery of high local antimicrobial concentrations using ALBC is needed. Most relevant clinical data showing the efficacy of ALBC for PJI prophylaxis and treatment are reviewed, and we dissected the limitations on the basis of the recent findings from animal models and suggested that aminoglycosides, in particular, could not be the best option. One of the major concerns associated with ALBC is the emergence of resistance because of theoretical prolonged exposure to low antibiotic concentrations. We summarize the mechanisms for the selection of resistant microorganisms, and we critically reviewed the evidence from animal models and clinical data from observational and registry studies and concluded that there is no evidence to support this association.

**Conclusion:**

While waiting for better evidence from well-designed clinical trials, ALBC shows a beneficial effect as a prophylaxis in arthroplasty, and to avoid the colonization of spacers used for two-stage revision in patients with PJI. Experimental models and clinical evidence suggest the need to achieve high local antimicrobial concentrations to obtain the highest prophylactic and therapeutic effect. The current evidence does not support the risk of increasing resistance with use of ALBC. In the future, it is necessary to evaluate new carriers and different antimicrobials to improve clinical outcomes.

## Introduction

Foreign-body infections are characterized by the presence of microorganisms in phenotypes that are different from the ones we encounter in other infections like urinary tract or respiratory tract infections. In the second one, microorganisms are actively replicating, induce a potent inflammatory response and can evolve to sepsis or septic shock in a few hours or days. In this scenario, prompt antibiotic treatment is necessary to reduce the mortality rate and, in general, it suffices to serve the purpose with a course of 7 days even in the presence of bacteremia [1]. In contrast, the presence of a foreign body (orthopaedic implant or necrotic/dead bone) changes the natural history of infection. Bacteria and fungi in specific environments surrounding implants (*e*.*g*., low pH, low oxygen or nutrient availability) are able to evolve, reducing their metabolism and virulence and forming small colony variants (SCV). This phenotype will be eliminated by phagocytic cells, but they protect themselves from immunity and antimicrobials in different ways [2]. Consequently, the clinical presentation of prosthetic joint infections (PJI) is more frequently chronic and their response to systemic antimicrobials poorer as compared to other infections, requiring prolonged courses (≥6 weeks) and implant removal to achieve high success rates [3].

According to this evidence, many years ago emerged the concept of using antibiotic-loaded bone cement (ALBC) to fix implants and reduce the number of infections or to make spacers to treat PJI, among other indications. Recently, excellent revisions about ALBC [4–6] have been reported and the present article aims, in general, to review the principles and limitations of ALBC on the basis of recent advances and, in particular, discuss its potential association with antimicrobial resistance. This paper is a narrative review and covers 5 main topics: local antimicrobials for the treatment of PJI, pharmacokinetics and efficacy of antibiotic-loaded polymethyl methacrylate, clinical evidence in infection prophylaxis using ALBC and PJI treatment using antibiotic-loaded spacers, resistance after exposure to polymethyl methacrylate loaded with antibiotics and definition of antimicrobial susceptibility to locally-delivered antibiotics.

## Are local antimicrobials necessary for the treatment of PJI?

The failure rate in PJI even after implant removal using a two-stage revision approach varies from 46 to 100% [7]. Although different reasons are proposed to explain the high failure rates, including mechanical problems related to the spacer or medical problems associated with protracted inactivity of the patient, the infection-related failures remain important.

**Fig. 1:**
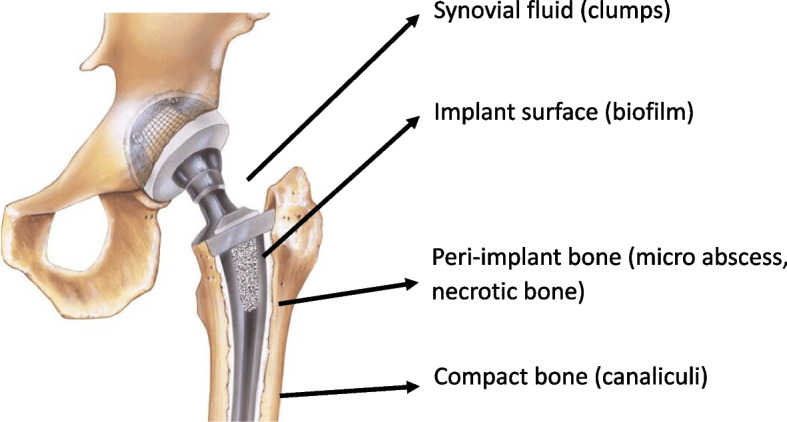
Potential structures that serve *S. aureus* small colony variants to protect themselves from immune system and antibiotics (see text for description)

Classically, it has been believed that a concentration of the antibiotic 100 to 1000 times the minimum inhibitory concentration (MIC) is necessary to eradicate biofilms [8]. This concentration is not achievable with systemically-administered antibiotics and consequently the standard treatment of chronic PJI includes the complete removal of the implant, cement, and necrotic/dead bone [9] and local delivery of high-concentration antimicrobials. The most widely used surgical strategy is the two-stage procedure using spacers of polymethyl methacrylate (PMMA) bone cement serving as antibiotic carriers. However, considering that adequate surgery removes the inert surfaces where the biofilm resides, is it still necessary to provide local antibiotics? In general, it is thought that there could be a residual biofilm. However, the presence of these structures has not been found in the peri-implant tissues. A question presents itself: Why the failure rate remains so high? We have been focusing on the presence of biofilms for many years, but probably the biofilm is just one of the placements where microorganisms remain protected. The bone and joint environment promotes the formation of SCV as an adaptive bacterial strategy to survive that is only apparent under stressful conditions, but not detected under the standard conditions under which MIC is measured in microbiology laboratories [10]. This bacterial phenotype is tolerant to antibiotics, but it would be eliminated by phagocytes if it were not for their ability to protect themselves. Beyond biofilms, other locations where SCV are protected include the clumps induced by synovial fluid, the intracellular space of osteoblasts, the abscesses in the bone surrounding implant, or the bone canaliculi (Fig. 1). Synovial fluid causes a staphylococcal aggregation (clumps) and it has been demonstrated that the concentration needed to eradicate these bacteria far exceeds the MIC of anti-staphylococcal cephalosporins [11]. Another location is the intracellular space and several *in vitro* experiments have described the ability of *Staphylococcus aureus* SCV to survive within osteoblasts [12], although no *in vivo* data have proven this potential mechanism. Other locations where SCV can persist has recently been described using a pig model that allegedly could define the histological and metabolic characteristics of the bone area surrounding an infected implant [13]. The model consisted of a small steel implant inserted in the tibial bone and inoculated with a low-inoculum of *S. aureus*. The main histological findings were: (1) the presence of abundant neutrophils forming microabscesses containing bacteria, and (2) vessel-free foci that consisted of osteonecrosis, inflammatory cells, bacteria, and bands of collagen. Both environments stimulated the formation of SCV and were good substrates for protecting SCVs from host immunity [14]. Finally, a recent experimental model and human biopsies from peri-implant infections have found *S. aureus* living in the bone canaliculi of compact bone adjacent to the infected implant [15–17]. Compact bone has bone canaliculi that consist of small channels from the lacunae (osteocytes) to the haversian canal (blood vessels) to provide passageways through the hard matrix. This space is used by *S. aureus* to escape from neutrophiles but, at the same time, the low vascularization of compact bone may predict that, in these canaliculi, the antibiotic concentration after its systemic administration is low. Consequently, it is reasonable to use local antimicrobials that theoretically can provide higher local concentrations to kill SCV. However, whether ×100-1000 MIC is enough (as for biofilms) or even higher concentrations are necessary to eradicate SCV in other locations is not well defined as well as the total exposure (days, weeks, months). It is of note that the capacity to form SCV and survive in different environments has been described mainly for *S. aureus* [18], and more data are needed for other pathogens.

## Pharmacokinetics of polymethyl methacrylate (PMMA) antibiotic elution

PMMA is an insoluble-in-water plastic that polymerizes from mixing a powder containing microspheres of PMMA and a polymerizing monomer in an exothermic reaction. Its insolubility does not permit the delivery of any water-soluble drug (antibiotics) contained within the PMMA and, theoretically, only the drug located in the surface of PMMA dissolves in the surrounding fluid. However, PMMA has intrinsic porosity due to the entrapment of air not completely removed during the polymerization. Although porosity reduces the strength of PMMA, this is a positive feature for drug delivery. PMMA porosity allows for fluid penetration and release of deep drug by dissolution and diffusion. Accordingly, the use of poragens (substances that increase the porosity of PMMA) has been evaluated to increase the drug delivery.

The kinetics of antibiotic elution is characterized by a first burst that represents the immediate delivery of the drug located on the surface of the PMMA, followed by the release of the drug within the PMMA because of fluid absorption into PMMA pores. The diffusion of the drug from the pores to the fluid surrounding the PMMA is directed by a concentration gradient. To quantify the total mass of antibiotic that is released from PMMA and to compare the eluent efficacy of different preparations or different antibiotics, the fluid in contact with PMMA is exchanged constantly over a period of 30 days when the release falls to near zero. This laboratory test maintains a low antibiotic concentration around the PMMA, simulating an infinite sink condition. These studies allow us to know the total antibiotic mass that can be released from PMMA and to study the parameters that predict the elution. From these studies, we know that the 3 main drivers of elution are (1) the molecular interaction between the antibiotic and the PMMA (in general not a problem except in some specific cases, such as anphotericin B that binds to PMMA and is not released, although this is overcome with liposomal formulation), (2) the PMMA surface in contact with the surrounding fluid, and (3) whether PMMA is subjected to loading or not.

The surface of PMMA can be modified by increasing the porosity. The antibiotic powder itself is a poragen and the size of the antibiotic particulates, the presence of water-soluble excipients (*e*.*g*., cyclodextrin in voriconazole preparation) and the total antibiotic dose per batch of PMMA will increase the porosity, thus leading to a higher percentage of antibiotic release at the expense of reduced strength of PMMA [19]. The percentage of antibiotic release using low doses is 3–5%, but this rate increases to 30–75% at high doses. A second determinant of the porosity is the mixing method. Mixing under vacuum reduces the air bubbles and, consequently, the porosity and increases the strength of the PMMA. At low dose (≤2 g per 40 g of PMMA), antimicrobials do not affect the PMMA polymerization and the mixing method does not alter significantly the drug release [20]. However, mixing under a vacuum reduces the release of antibiotics when high doses (≥10 g per 40 g of PMMA) are used [21]. Orthopedic surgeons should be aware that, with high doses, hand mixing is better for elution purposes but significantly reduces the strength of the PMMA and a metal core is recommended. In addition, using more than 16 g of antibiotics per 80 g of PMMA makes mixing difficult in spacer molding [22]. Finally, spacers subjected to loading, like daily activities, increase the antibiotic release up to 2 times [23].

The efficiency of PMMA to elute a specific antibiotic *in vitro* is important, but we need to know the concentration achieved in the surrounding tissue, synovial fluid, and peri-implant bone for prophylactic and therapeutic purposes (Fig. 1). The dose used in prophylaxis is 0.5–2 g per 40 g of PMMA and the mantle applied around the prosthesis is few millimeters thick. We don't know the concentration achieved in the peri-implant space, but the small fluid volume and the limited flow in the implant/bone interface predicts high levels. On the other hand, the data from postoperative drain fluid levels reflect the antibiotic concentration that protects the extraosseous parts of the implant. These reports showed a peak at 12 h that generally is above the MIC of susceptible microorganisms, but then the concentration decreases rapidly. Considering that most implant infections are the consequence of intraoperative contamination, the use of local antibiotics could help the systemic one to reduce the risk of infection. In addition, large national registries with long follow-up support that no reduction in the implant fixation occurs when antibiotic-loaded cement is used and even aseptic loosening is less frequent [24]. However, there are concerns about the potential association between antimicrobial resistance and whether aminoglycosides continue to be the best agents for prophylaxis. These aspects are addressed in the following sections.

For therapeutic purposes, antibiotic-loaded spacers have been widely used in a two-stage exchange procedure. Antimicrobial delivery from spacers have been analyzed by measuring concentrations in the postoperative drain fluid [25–27]. From these studies the major conclusions are: (1) the higher the dose and the number of antibiotics in the cement, the higher the antibiotic elution, (2) doses of > 3 g of antibiotic per 40 g of PMMA deliver concentrations higher than 500 mg/L during the first 7 days after spacer placement, (3) concentrations well above the MIC of microorganisms involved in PJI remain at the moment of spacer removal (>6 weeks), (4) doses of 1–4 g of antibiotic per 80 g of PMMA deliver concentrations below 50 mg/L at peak and around 1–5 mg/L after 1 week, and even lower concentrations at the moment of implant removal, and (5) systemic antibiotic concentrations, even at high local doses, are only < 2 mg/L.

This data suggest that by using spacers with high doses, concentrations >100 times the MIC of the microorganisms causing PJI can be achieved. However, all the information comes from studies using drain fluid. The problem is that there are no data showing the concentrations in the peri-implant bone and compact bone surrounding the infected implant. This is important since data suggest that the elution from spacers is not homogenously distributed [28], and transcortically-transported (from peri-implant tissue to cortical bone) antibiotic is sub-optimal [29]. To learn more about the potential usefulness and limitations of local antibiotic delivery using PMMA, we are going to review the most recent and relevant information coming from experimental models that best mimic the PJI conditions.

### Efficacy of antibiotic-loaded PMMA for the treatment of PJI in experimental models

Experimental models of PJI have been established using different materials, locations, or animals. *Staphylococcus aureus* is the most used pathogen, but the microorganism and the material are pre-incubated and then implanted in some cases, or the pathogen is inoculated before or after the implant placement in others. This heterogenicity makes it difficult to extend the efficacy of different interventions to humans [30]. Several models have evaluated the efficacy of antibiotic-loaded cement as prophylaxis, but few have been designed to analyze its efficacy as treatment of PJI using antibiotic-loaded cement spacers. Carli *et al*. [31] made a mouse model of two-stage revision for PJI caused by *S. aureus* using an antibiotic-loaded cement spacer. A 40 g of PMMA powder was mixed with 2 g of vancomycin powder by using a spatula. The mixture was used to form knee spacers. Twenty mice underwent proximal tibial implantation with 3 × 10^5^ colony-forming units (CFUs) of *S. aureus* and after 2 weeks, and 9 were randomly selected for receiving revision surgery. An articulating vancomycin-loaded PMMA spacer was inserted into the proximal tibia and the animals were followed for 6 weeks without systemic antibiotics. At the end of the follow-up, animals treated with spacers had lower levels of inflammatory biomarkers in comparison to their non-treated counterparts, and no signs of periosteal reaction or proximal tibial fragmentation was found on X-ray and no purulent material during surgery was present in non-treated ones. The total amount of CFU from implant sonication was significantly lower in vancomycin-loaded spacers than in the original infected implants. In contrast, the culture of periprosthetic tissue counts were similar in treated (2.53 ± 3.2 × 10^5^ CFUs) and non-treated (3.7 ± 3.38 × 10^5^ CFUs, *P* = 0.261) mice. This study suggests that antibiotic-loaded cement spacers without systemic antibiotics are effective for preventing spacer surface bacterial colonization and reducing the soft-tissue damage, but fail to eradicate *S. aureus* from the periprosthetic tissue, at least when dosage was at 2 g in 40 g of PMMA.

### What happens in the periprosthetic tissue of an infected implant that even high local antibiotic concentrations are not enough to eradicate the infection from the peri-implant tissue?

To answer this question, it is of interest to come back to the above-mentioned pig model developed by Jensen *et al*. [13]. The histological characteristics showing microabscesses and free vascular areas make it difficult for the antibiotic diffusion to attain the target. Indeed, they observed low gentamicin concentrations (<3 mg/L) in the bone adjacent to gentamicin-loaded calcium sulphate in the pig model [32]. This low gentamicin concentration associated with the documentation that the peri-implant bone had low oxygen concentration [33] could explain the limited therapeutic efficacy of aminoglycosides. It is of note that aminoglycosides require oxygen to cross the bacterial cell membrane, and consequently, their activity in hypoxic environments is limited and they are non-active against anaerobes. These findings call into question the role of aminoglycosides in the treatment of peri-implant osteomyelitis, while they retain a good efficacy as local antibiotic prophylaxis.

In summary, local antibiotics are needed but the presence of microabscesses and bone necrosis in the peri-implant bone area together with a hypoxemic environment significantly lower the concentration of the antibiotic in the peri-implant tissue and also reduce the efficacy of antibiotics, but, in particular, aminoglycosides. New carriers delivering higher antibiotic concentrations and different antibiotics should be evaluated in the future. In the next sections we review the clinical trials assessing the efficacy of antibiotic-loaded PMMA for reducing the infection rate in joint arthroplasty and the evidence for the treatment of PJI using antibiotic-loaded spacers.

### Clinical evidence in infection prophylaxis using ALBC

The role of ALBC in PJI prophylaxis after total joint arthroplasty has been widely discussed. Several systematic reviews and meta-analyses have recently analyzed the current evidence of the effect of ALBC *vs.* plain bone cement (PBC). Sultan *et al*. [34] showed contradictory results for PJI rates when using ALBC in primary procedures in comparison with PBC, while Sebastian *et al*. [35] supported its effectiveness in preventing PJI in total joint arthroplasty. Ekhtiari *et al*. [36] reviewed five randomized controlled trials that showed a potential benefit of ALBC in both primary and revision arthroplasty surgery, but with no statistically significant overall differences. Farhan-Alanie *et al*. [37] found a protective effect against infection in total hip arthroplasty (THA), but not in primary knee replacements. On the opposite, Xu *et al*. [38] defended the potential reduction of PJI after primary total knee arthroplasty (TKA) by using ALBC, especially with the use of gentamicin and high-dose ALBC (≥1 g/40 g of PMMA). Some studies have also shown a reduction in aseptic loosening when using ALBC, with a lower all-cause revision rate, probably because of undiagnosed PJIs [39–41], while other authors did not support this postulate [42]. Despite these variable results, we must remark on the huge heterogeneity of the reported meta-analyses. Table 1 summarizes the randomized controlled trials (RCT) that compared ALBC and PBC [43–46], excluding first experiences from Pfarr *et al*. and Wannske *et al*. for lacking data [47, 48]. Three articles showed benefit, and the other 2 did not, but one yielded a low PJI rate (1%) and the other used colistin and erythromycin as local antibiotics with poor activity against Gram-positive microorganisms. Chiu *et al*. had also demonstrated the benefit of ALBC in higher risk population, such as patients with diabetes mellitus with a previous smaller-sized RCT in which cefuroxime-impregnated cement prevented PJI (0% in ALBC-group *vs.* 13.5% in PBC-group, *P* = 0.021) [49].

**Table 1:** Randomized controlled trials comparing ALBC *vs*. PBC in PJI prophylaxis during total joint replacement

First Author, year	Country	Study period	Antibiotic, dose (g/40 g PMMA)	Joint	*n* (ALBC/PBC)	PJI ALBC *n* (%)	PJI PBC *n* (%)		Comments
Josefsson *et al*. 1981 [43]	Sweden	1976–1978	Gentamicin, 0,5	Hip	1633 (821:812)	3 (0,37)	13 (1,60)	*P* < 0.01	No systemic prophylaxis in ALBC group
McQueen *et al*. 1990 [44]	UK	1982–1985	Cefuroxime, 1,5	Hip and knee	401 (201:200)	2 (1,00)	2 (1,00)	n. s.	No systemic prophylaxis in ALBC group
Chiu *et al*. 2002 [49]	China	1994–1998	Cefuroxime, 2	Knee	340 (178:162)	0 (0,00)	5 (3,09)	*P* 0.0238	
Chiu *et al*. 2002 [49]	China	1993–2004	Vancomycin, 1	Knee	183 (93/90)	0 (0,00)	6 (7,00)	*P* 0.0130	
Hinarejos *et al*. 2013 [46]	Spain	2005–2010	Erythromycin, 0,5/ Colistin 3 MU	Knee	2948 (1483:1465)	20 (1,35)	20 (1,37)	n. s.	

Consistent with the conclusion of a meta-analysis by Xu *et al*. that higher antibiotic doses yielded better results, Sprowson *et al*. [50] demonstrated better outcomes with the use of high-dose dual-antibiotic local prophylaxis in comparison with low-dose single antibiotic. This study was not included in most meta-analyses because it did not incorporate a PBC arm, but the rate of PJI was 1.1% in the clindamycin 1 g plus gentamicin 1 g group against 3.4% in the 1 g single-gentamicin group (*P* = 0.041). Tyas *et al*. [51] showed that this effect was maintained over time, and it was even associated with a lower number of resistant strains in infected cases (although the proportion of infection with resistant microorganisms were higher in this group in relative terms as discussed later). As we pointed out before, doses up to 2 g/40 g of PMMA do not alter the biomechanical properties of the cement and may confer benefits in prophylaxis.

Regarding observational retrospective studies, they have the main limitation of selection bias. Higher-risk patients are more likely to receive ALBC, so we can expect worse outcomes in large series despite control for confounding factors. This issue has been proved in some studies, where there was a significantly higher presence of comorbidities in the ALBC group than in the PBC group: more diabetes, higher ASA grade and more indications different from osteoarthritis [52], more severe obesity, more bilateral surgeries, and longer surgery duration [53] or more cases of diabetes, obesity, hepatitis C virus infection, higher smoking rates, and greater ASA grades and charlson comorbidity index (CCI) scores [54].

Furthermore, the outcome chosen in most of the studies (revision rate for PJI) may underestimate the actual PJI rate and the potential benefit of ALBC in the prevention of early infections, as many of these patients do not undergo a revision procedure but a debridement with implant retention, and national registries do not encode debridement as revision surgery, neither they collect conservative treatments with antibiotic suppressive therapy. Habitual concerns about the use of ALBC, such as emerging of antibiotic-resistant microorganisms, do not represent a limitation as discussed later in this document.

In conclusion, there is an important lack of high-quality evidence about the efficacy of ALBC in primary prophylaxis of PJI. However, while expecting results of ongoing prospective studies, such as ALBA trial [55], we believe that 2 g of antibiotic (gentamicin) or a combination of 1 g of 2 antibiotics per 40 g of PMMA in patients with risk factors for infection is a reasonable strategy. Further randomized controlled studies should be carried on.

### Clinical evidence in prosthetic joint infection treatment using antibiotic-loaded spacers

In 2012, Iarikov *et al*. [7] reviewed the experience with antibacterial cement spacers in 2-stage hip or knee arthroplasty for the treatment of PJI. Their conclusion was that data published do not allow a decision to be made on the best antibiotic and the adequate dose. Therefore, we have reviewed the new literature from 2012 to explore potential advances that help to clarify the role of antibiotic-loaded spacers.

We searched for those articles that describe the reinfection rate in more than 10 PJI treated with 2-stage revision surgery using antibiotic-loaded cement spacers and they provide information about the total dose of the antibiotic in the cement. High dose of local antibiotic was considered when ≥ 2 g was used or when ≥ 2 antibiotics were mixed disregarding the individual dose. A total of 7 articles were retrieved, with 6 having the outcome of re-infection after the second stage [56–61], and 1 giving the culture result at the second stage [62]. The main characteristics of the articles are depicted in the Table 2. The eradication rate using low antibiotic dose ranged from 57 to 81%, although considering only those studies that evaluated infection eradication it was from 80–81%. The rate for those cases receiving high antibiotic dose ranged from 60 to 98%. The 60% eradication rate was observed in 10 cases infected with methicillin-resistant *S. aureus*, but upon removal of this group, the rate varied between 79 and 98%. From these results, it was difficult to reach any firm conclusions, although it seems that higher eradication rates were associated with high doses. On the other hand, the study from Carvalho *et al*. showed that combination of 2 antibiotics in the cement spacer was associated with significantly lower rate of positive cultures during the second stage. These results are in line with the findings reported by Wouthuyzen-Bakker *et al*. [63] in 344 cases and specifically showed that adding vancomycin to gentamicin cement spacers significantly reduced the isolation of coagulase-negative *Staphylococci* in the second stage. Therefore, these studies support the need to select an active local antibiotic and use high doses. However, we need more studies to evaluate the efficacy of local antibiotics and whether their use modifies the application and duration of systemic ones.

**Table 2:** Articles describing the infection eradication rate using antibiotic-loaded spacers

First Author, year, (country)	N° of joints (N patients)	Joint	Type of spacer (N)	Antibiotic, total dose (amount^**a**^)	Rate (%) of reimplantation	Infection eradication rate (%)
Radoicic *et al*. 2016 [56] (Serbia)	18	Knee	Handmade	VAN 2 g (high)	67	89
				ERT 4 g (high)		
				CZA 2–4 g (high)		
Dias Carvalho *et al*. 2021 [62] (Portugal)	58	Hip and Knee	Commercial G (14)	GEN (low)	100	57^b^
			Commercial G-V (24)	GEN + VAN (high)		
			Handmade (20)	VAN 3 g + MER 2 g + GEN 0.5 g (high)		89^b,c^
Ortola *et al*. 2017 [57] (Italy)	112	Knee	Handmade	CLI 1 g + GEN 1 g (high)	75	96
				CLI 1 g + GEN 1 g + VAN 4 g (high)	92	98
Vasarhelyi *et al*. 2022 [58] (Canada)	176	Knee	Handmade	VAN 2 g and TOB or GEN 2.4 g (high)	NA	87
Corona *et al*. 2013 [59] (Spain)	46 (41)	Hip and Knee	Commercial G (20)	GEN 0.8 g to 3.2 g (low)	100	80
			Commercial G + V (26)	VAN + GEN (1:1) 0.8g to 3.2g (high)	88	85
Uchiyama *et al*. 2013 [60] (Japan)	37 (36)	Hip	Handmade	GEN 1.2 g (low)	86	81
				GEN + VAN 2 g if MRSA (high)		60
Nodzo *et al*. 2017 [61] (USA)	140	Knee	Commercial (58)	VAN 1 g + AG 3.8 g (high)^d^	140 (100%)	83
			Molds (43)	VAN 2.7 g + AG 4.4 g (high)^d^		88
			Tibial mold femoral autoclaved (39)	VAN 2 g + AG 2 g (high)^d^		79

*VAN* Vancomycin, *ERT* Ertapenem, *CZA* Ceftazidime, *GEN* Gentamicin, *CLI* Clindamycin, *MER* Meropenem, *TOB* Tobramycin, *AG* Aminoglycoside

^a^For definition see the text

^b^Eradication evaluated as culture negative at second stage

^c^Eradication rate of both groups GEN+VAN or VAN+MER+GEN

^d^Median dose of each antibiotic

## Selection of resistant mutants after exposure to PMMA loaded with antibiotics

There is an ongoing debate regarding the risk of selection of resistant microorganisms because of the use of PMMA loaded with antibiotics. Before discussing the evidence from the literature, it is helpful to understand the basic concepts of antimicrobial resistance.

**Fig. 2:**
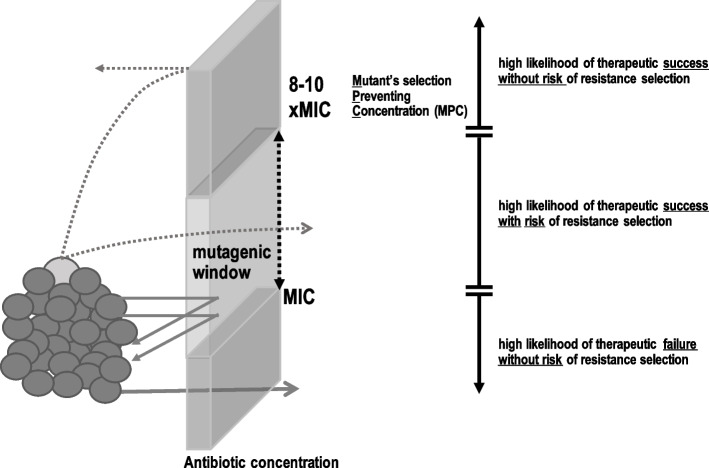
Descriptive definition of the mutant preventing concentration and mutagenic window

The acquisition of resistant microorganisms could be the consequence of (1) transmission of resistant pathogens from patient to patient via the hands of healthcare workers or directly inoculating the pathogen during invasive procedures (surgeries), or (2) the selection of the pre-existent resistant mutants in the infection foci or in the microbiota of patients after being exposed to antibiotics. The second mechanism of acquisition is the one potentially associated with the use of antibiotic-loaded bone cement. To know whether this risk exists, we summarize the pharmacodynamic principles that govern this selection process. First, it is important to remember that resistant bacteria spontaneously emerge because of DNA replication errors that are not repaired during bacterial cell division. Resistant bacteria remain in low numbers because they are not able to compete with their susceptible counterparts for nutrients. The specific number of resistant cells varies for each bacteria-antibiotic combination, and it can be as frequent as one mutant per million bacteria (*e*.*g*., *Staphylococci*-rifampin). The opportunity for resistant mutants to overgrow arrives when the bacterial population is exposed to antibiotics that, by killing the susceptible ones, reduce the competence. However, not all antibiotic exposures are selecting for resistant sub-population. When a bacterial population is exposed to sub-MIC concentrations, no selection of resistant bacteria occurs (Fig. 2). If the exposure is over the MIC but below the mutant-preventing concentration (MPC), the selection of resistance takes place, while concentrations over the MPC will avoid the selection (see Fig. 2 for definitions).

MIC is the minimal inhibitory concentration that is a measure used in all the microbiology laboratories to define the susceptibility pattern of the clinically isolated pathogenic bacteria. Mutant preventing concentration (MPC) is the MIC of the first pre-exposed resistant sub-population (light grey color). The concentrations ranging from the MIC to MPC are those significantly associated with resistance selection and are designated as mutagenic window.

According to the pharmacodynamic principles of resistance selection, it is reasonable to use antibiotic-loaded bone cement that delivers a high antibiotic dose and theoretically achieves antibiotic concentrations > 10×MIC in the surrounding tissues, which is clearly higher than the MPC that, in general, is 8–10×MIC. However, recent data from the National Joint Registry for England, Wales and Northern Ireland showed a higher proportion of gentamicin-resistant strains in PJI from primary arthroplasties performed with gentamicin-loaded cement [64]. The authors evaluated 166 PJI cases in whom the primary arthroplasty was performed with gentamicin-loaded cement and 49 uncemented cases. All were infected with staphylococci and the susceptibility pattern was available for review. In the first group, 59 out of 166 PJIs were caused by a gentamicin-resistant isolate (36%) and in the second group only 6 out of 49 (12%) had PJIs caused by the isolate. This difference was statistically significant (*P* = 0.001) and the multivariable analysis, after adjusting for patient and surgical factors, showed that gentamicin-loaded cement was associated with an eight-fold increase in the risk of gentamicin resistance (OR 8.3; CI95% 2.2–30.2, *P* = 0.001). Similar results were observed by Tyas *et al*. [51] in an analysis of the data obtained from a clinical trial comparing the prophylactic efficacy of cements loaded with gentamicin or with gentamicin plus clindamycin. As expected, the rate of gentamicin-resistant strains among infected patients was similar between both groups. However, this trial presented an opportunity to compare the impact of using or not using clindamycin. The rate of clindamycin resistance was significantly higher in those receiving local clindamycin (96%) than in those not receiving it (36%). Stefánsdóttir *et al*. [65] evaluated the PJI related to the Swedish knee arthroplasty registry where cement loaded with gentamicin is mandatory, and they also identified a significant increase in the gentamicin-resistant staphylococci, but no control was available. In contrast, similar analysis of a large database in the US did not find an increased risk of resistant pathogens when tobramycin was loaded into the cement of primary arthroplasties [66]. However, these authors evaluated the resistance to methicillin, tetracycline, and erythromycin but not to tobramycin. As a result, they could not confirm the risk of being infected with tobramycin-resistant isolates. This is an important concept because surgeons should be aware that when a revision surgery for a PJI is performed in a patient with a primary arthroplasty cemented with antibiotic, the same antibiotic should not be used in the spacer, when a 2-stage revision is the surgical option, or in the cement of the revision arthroplasty in the case of one-stage revision.

There are few studies about the increase in resistant strains using spacers loaded with antibiotics. The best analysis was performed by George *et al*. [67] because they retrospectively selected patients with the *S. aureus* isolated in the first and second stages of patients who underwent a 2-stage revision surgery using vancomycin-loaded spacers. The MIC increased (1–2 mg/L *vs.* ≤ 0.5 mg/L) in 9 out of 25 cases (36%). Interestingly, the authors evaluated 5 additional cases that received spacers without vancomycin and no one had an increase in the vancomycin MIC. Corona *et al*. [68] compared the resistant rate in cultures at the moment of removal of 52 gentamicin-loaded spacers and in cultures from 61 PJI without antibiotic-loaded spacer. The rate was significantly higher in the first group (49.2%) than in the second group (19.3%, *P* = 0.0001). However, this analysis should be interpreted cautiously since the resistant rate of the primary pathogens was not provided and so it is not possible to ensure that local antibiotic use was responsible for the increased resistance rate. In the future we need more quality data on resistance rate related to the use of spacers.

As clinical evidence supports the increase of resistance when using antibiotic-loaded cement, does this mean that clinical evidence is opposed to pharmacodynamic principles discussed above? There are two potential explanations to illustrate that there is no contradiction. The first one is that, as we described previously, the release of antibiotic from the PMMA is not homogeneous [69] and different ways to mix it result in different antibiotic release [70]. The consequence is that some bacteria are exposed to concentrations within the mutagenic window, and this could explain the increase in vancomycin MIC in the George *et al*. study [67]. A second explanation could be that patients are primarily infected during surgery by a pathogen highly resistant to the local antibiotic used (MIC higher than the local concentration achieved) and consequently the infection is not prevented [71]. Whatever scenario occurs, the local antibiotic fails to avoid the infection (prophylaxis) or to cure the infection (treatment) and now the cement becomes an excellent inert surface to facilitate the biofilm formation responsible for the bad evolution [72, 73]. This means that the selection of local antibiotic should be made in agreement with the local epidemiology for prophylaxis or the susceptibility pattern for infection.

**Fig. 3:**
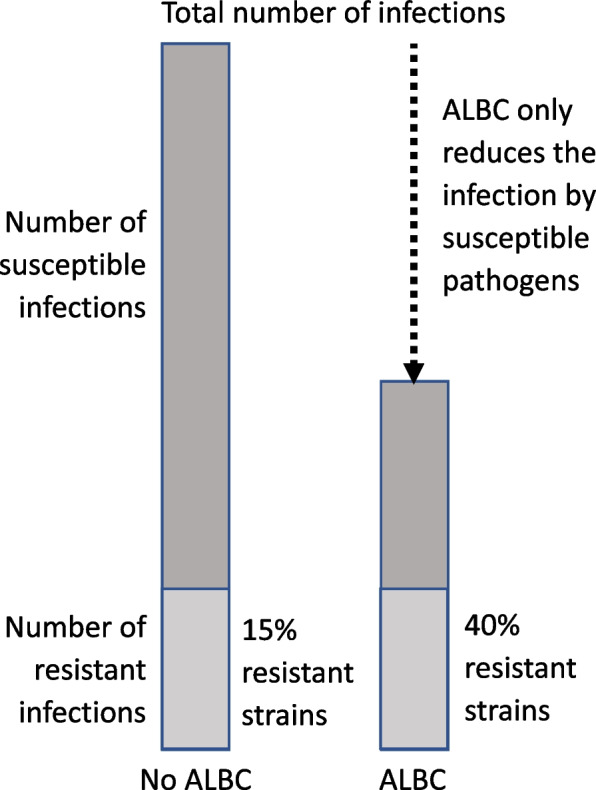
Illustration of the impact of antibiotic-loaded bone cement (ALBC) on the total number of infections and the consequent final percentages of susceptible and resistant pathogens (considering 100% prophylactic efficacy of local antibiotics)

Given these explanations, the interpretation of increasing resistance using local antibiotics should be adequately addressed. The total number of infections when local antibiotics are not used is higher and only a small percentage of them (*e*.*g*.< 10%) are resistant. In contrast, if we assume a high effectiveness of local antibiotics in reducing the number of infections caused by susceptible pathogens, the number of infected cases will be lower, but these infections will be caused by resistant pathogens and the relative rate of resistance will be much higher, leading to a misinterpretation of a significant increase of resistance (Fig. 3). This concept was well illustrated by Thornes B *et al*. [74] using an animal model with gentamicin pellets infected with *Staphylococcus epidermidis* as well as in the previously discussed experience from a clinical trial using or not using local clindamycin [51]. In the future, when the authors report the risk associated with the use of local antibiotic bone cement as prophylaxis, they should clearly state the absolute numbers and rate of resistance with and without the exposure.

On the other hand, one of the major concerns on antibiotic-loaded cement and resistance is the potential risk of concentrations below the MIC for several days or weeks. Many systemic antibiotics are partially eliminated through the biliary tract, and they achieve enough concentrations in feces to reduce the total amount of healthy microbiota. The consequence is dual: It creates an ecological void that permits the overgrowth of resistant minor sub-populations present in the microbiota, and it makes the host more susceptible to colonization by environmental resistant bacteria. The low systemic concentrations of gentamicin or vancomycin from bone cement, and the renal elimination of both drugs support the idea that they have a minor impact on gut microbiota. This reinforces the opinion of the authors that, with the current knowledge, antibiotic-loaded cement is useful for prophylaxis and the risk of selecting resistance is low, but it fails when microorganisms contaminating the wound during surgery are resistant to the local antibiotic. In the future, and according to the local epidemiology, it will be necessary to re-evaluate whether aminoglycosides should still be the best option for prophylaxis.

### Break-points for defining antimicrobial susceptibility to locally-delivered antibiotics

**Fig. 4:**
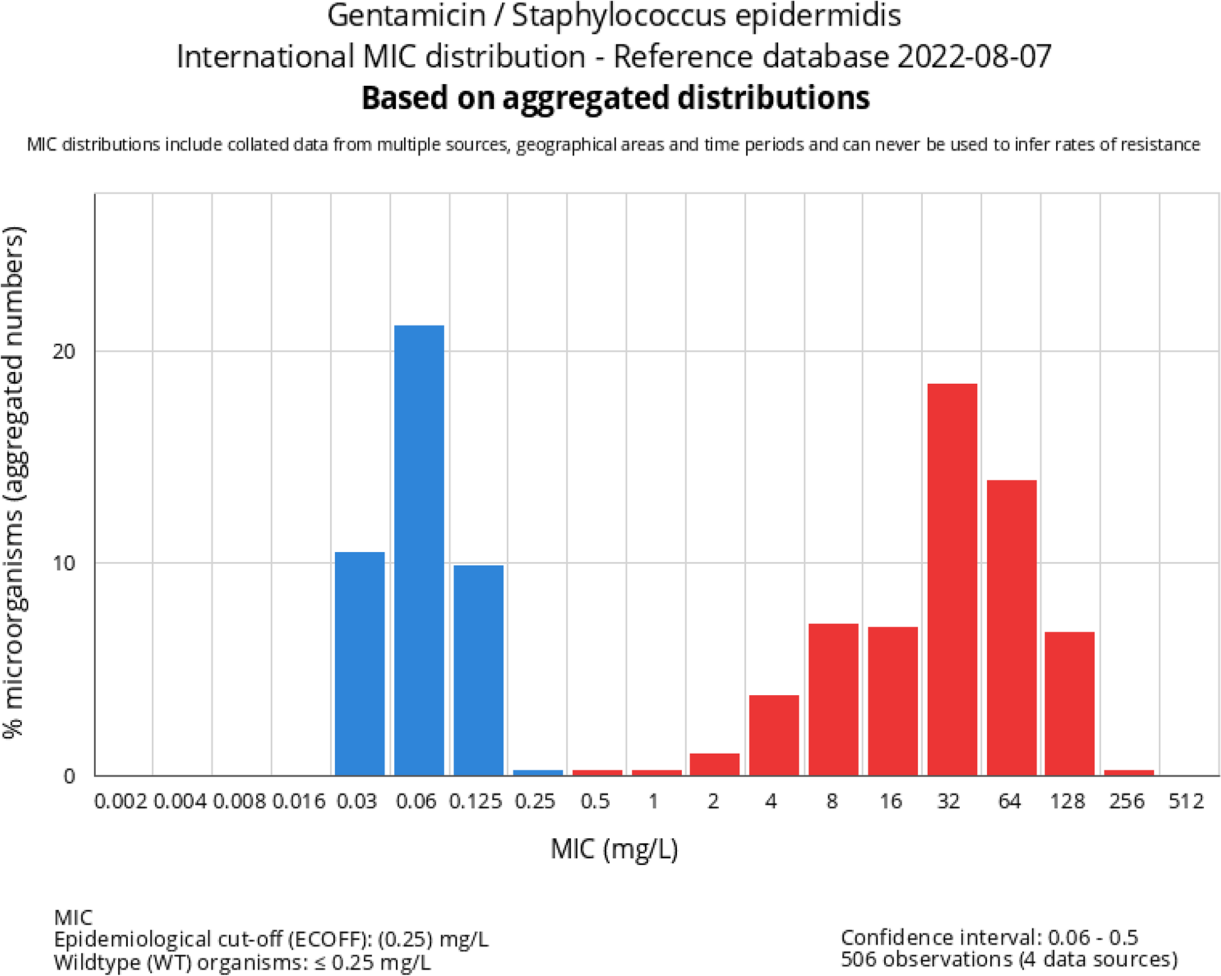
Gentamicin MIC distribution for *S. epidermidis* from 506 observations. The break-point for susceptible staphylococci for systemic administration of gentamicin is ≤ 2 mg/L. A potential break-point for local gentamicin delivery, based on the data about the elution from ALBC and *in vitro* data (see text), could be > 32 mg/L (source of the figure: https://mic.eucast.org, data August 6th, 2022).

An open question is whether the break-points to define susceptibility should be the same for local and systemic antibiotics. The break-points established by international agencies (CLSI or EUCAST) are based on the serum concentrations potentially achieved with systemic administration (*e*.*g*. gentamicin MIC for susceptible staphylococci ≤ 2 mg/L). However, the concentrations locally obtained from antibiotic-loaded bone cement are significantly higher and *in vitro* data showed that coagulase-negative strains with a gentamicin MIC ≤ 32 mg/L were effectively eliminated by gentamicin-loaded cements. This was not the case for strains with an MIC ≥ 256 mg/L [71] so the point to differentiate between susceptible and resistant strains to local gentamicin should be somewhere between 32 and 256 mg/L. This explains why although the resistant rate of staphylococci to aminoglycosides is high, they remain potentially useful local antibiotics for prophylaxis. Considering that the range of gentamicin MIC is from 0.03 to 256 mg/L (mic.EUCAST.org webpage), in the future it will be helpful to precisely monitor the MIC for aminoglycosides and to adapt the breakpoints for local antibiotics (proposal in Fig. 4). In contrast to aminoglycosides, vancomycin MIC range is significantly narrower, and it remains < 32 mg/L for staphylococci (mic.EUCAST.org webpage). Therefore, this antibiotic seems more appropriate for prophylaxis and treatment than aminoglycosides, particularly, now that many effective alternatives to glycopeptides are available (*e*.*g*. daptomycin, linezolid, dalbavancin, ceftaroline) and thus the concern about vancomycin resistance is significantly lower. Future randomized controlled trials should be proposed to answer this question.

## Conclusion

While waiting for better evidence from well-designed clinical trials, ALBC shows a beneficial effect as prophylaxis in arthroplasty, and to avoid the colonization on spacers used for two-stage revision in patients with PJI. Experimental models and clinical evidence suggest the need to achieve high local antimicrobial concentrations to obtain the highest prophylactic and therapeutic effect. In the future, it is necessary to evaluate new carriers and different antimicrobials to improve the clinical outcomes.

## Data Availability

All data generated or analyzed during this study are included in this published article and its supplementary information files.

## Abbreviations

ALBC: Antibiotic-loaded bone cement
PJI: Prosthetic joint infection
SCV: Small colony variant
MIC: Minimum inhibitory concentration
PMMA: Polymethyl methacrylate
CFU: Colony-forming unit
PBC: Plain bone cement
THA: Total hip arthroplasty
TKA: Total knee arthroplasty
RCT: Randomized control trial
MPC: Mutant preventing concentration
OR: Odds ratio
